# Molecular mechanisms of exercise-induced improvements in Alzheimer’s disease: a focus on lipid homeostasis

**DOI:** 10.1186/s40035-026-00537-5

**Published:** 2026-03-23

**Authors:** Jianfan Zhou, Xianliang Zhang, Shuting Yin, Shuan Xue, Qiang He, Si Chen, Xiangli Xue

**Affiliations:** 1 School of Physical Education, Shandong University, 17922 Jingshi Road, Lixia District, Jinan, 250061 China; 2 College of Health Sciences, Shandong University of Traditional Chinese Medicine, Jinan, 250355 China; 3 School of Nursing and Rehabilitation, Cheeloo College of Medicine, Shandong University, Jinan, 250102 China

**Keywords:** Exercise, Alzheimer’s disease, Lipid, Lipid homeostasis

## Abstract

Alzheimer’s disease (AD) is the most prevalent type of dementia, and its pathophysiological mechanisms involve multiple factors, including genomic factors, metabolomic factors, and environmental factors. Lipid dysregulation occurs both centrally and peripherally in patients with AD, and the severity is closely associated with disease progression. Applied studies based on genome-wide association studies, genomic analyses, lipidomic analyses, mass spectrometry, and machine learning, have identified lipids as a key potential target for early diagnosis and intervention in AD. However, due to the complexity of AD pathogenesis and the considerable structural and functional diversities of lipids, pharmacological therapies that target lipid homeostasis have shown limited effectiveness in ameliorating AD pathology and are often accompanied by side effects. In contrast, exercise, a holistic intervention with multitarget effects, can modulate the levels of multiple lipids simultaneously and slow the progression of AD with minimal side effects. However, the mechanisms require further clarification. This review summarizes alterations and mechanisms of action of lipids—including fatty acids, triglycerides, glycerophospholipids, sphingolipids, and cholesterol—in AD and further outlines the possible molecular mechanisms through which exercise influences AD through modulation of lipid metabolism. We also review the recent clinical research on lipid-targeting drugs for AD, and propose a hypothesis that lipids may act as a mediator of the peripheral–central crosstalk between exercise and AD. Additionally, how different apolipoprotein E genotypes may affect the response to exercise in AD is explored. These insights provide a theoretical basis for nonpharmacological interventions for AD and offer an important reference for the development of lipid-related therapeutic targets.

## Introduction

Alzheimer’s disease (AD) is the most common form of dementia and one of the most prevalent neurodegenerative diseases among older adults, accounting for approximately 90% of dementia cases in this population [1]. According to the World Alzheimer Report 2019 published by the World Health Organization (WHO), about 24 million people worldwide are currently living with AD, and this number is projected to quadruple by 2050 [2,3]. The primary pathological features of AD include amyloid plaques resulting from the extracellular accumulation of β-amyloid (Aβ), and neurofibrillary tangles (NFTs) formed by intracellular aggregation of hyperphosphorylated tau protein [4]. In addition to these hallmark features, several other pathological processes, including neuroinflammation, glucose metabolism dysregulation, mitochondrial dysfunction, oxidative stress, and lipid disturbances, have been implicated in the pathogenesis of AD [5–7].

A large body of clinical and basic research has confirmed that the dysregulation of lipid homeostasis is a risk factor for AD, closely associated with the onset and progression of the disease. Notably, as early as 1907, the first neuropathological features of AD identified by Dr. Alois Alzheimer involved lipid abnormalities. In recent years, studies have further demonstrated that the dysregulation of lipid homeostasis is a consistent pathological feature in AD patients and AD mouse models. Conversely, restoring lipid homeostasis improves cognitive function in both AD patients and animal models [8]. Moreover, with continued advancements in technology, applied research using various approaches—including genome-wide association studies, genomic analyses, lipidomic analyses, mass spectrometry, and machine learning—has identified lipids as promising biomarkers for AD [9,10].

To date, several drugs have been approved for the treatment of AD. However, due to the inherent side effects and the long development timelines of pharmacological therapies [11], an increasing number of researchers have switched focus to nonpharmacological interventions [12]. Regular physical exercise has been shown to prevent and delay the progression of AD by regulating oxidative stress, inflammation, and metabolism, with minimal side effects [13]. Notably, regular exercise may hold greater potential than pharmacotherapy for maintaining lipid homeostasis in the AD brain. The multitarget effects of exercise are more strongly aligned with the complex and multifaceted nature of lipids, potentially resulting in integrative benefits superior to those of pharmacotherapy. Moreover, regular exercise may be particularly relevant for maintaining lipid homeostasis during the early stages of AD. Additionally, patients are generally more capable of engaging in physical activity during the early stages of the disease [14]. Previous studies have demonstrated that exercise can reduce Aβ deposition and abnormal tau phosphorylation by regulating brain lipid homeostasis in both AD patients and animal models, thereby improving learning, memory, and overall cognitive function [15,16]. Although several studies have specifically examined how exercise improves AD through the restoration of systemic lipid homeostasis, few have reviewed the molecular mechanisms through which exercise influences AD via lipid regulation. In this review, we summarize the associations between various lipids and AD, elucidate the mechanisms through which exercise modulates lipid homeostasis to influence AD progression, and propose a hypothesis that lipids may act as a mediator of the peripheral–central crosstalk during exercise in AD. Additionally, how different apolipoprotein E (*APOE*) genotypes may affect the response to exercise in AD is explored. This paper provides a reference for clarifying the relationships among exercise, lipids, and AD.

## Lipids and AD

Lipids are the main components of cell membrane and play essential roles in regulating physiological processes such as membrane synthesis, energy supply, and cellular signaling [17]. Lipid membranes have been described as “eternal structures” [18]. Dysregulation of lipid homeostasis is associated with the onset, progression, and severity of various neurodegenerative diseases, including AD, Parkinson’s disease, and multiple sclerosis, affecting both the peripheral nervous system and the central nervous system (CNS) [19]. Table 1 summarizes changes in the levels of various lipids across different tissues as observed in clinical and animal studies of AD pathology.

**Table 1:** Changes in the levels of various lipids in AD

Subjects	Groups	Sample/tissue	Changes in AD	Analytical method	References
Human subjects	CH = 70	CSF	FFA↑	GC–MS	[20]
	MCI = 40	Supernatant fluid	DHA↓		
	AD = 29	NP	MUFAs (C15:1,C19:1)↑ PUFAs (C20:2n-6, C20:3n-3, C22:4n-6, C22:5n-3)↑		
Human subjects	CH = 15	Post-mortem neocortex region (Brodmann area 7)	DHA↑	GC–MS	[21]
			Elaidic acid↑		
	AD = 14		PA↑		
APP/PS1 double-transgenic mice	AD = 10	Serum	FFA↓	GC–MS	[22]
	WT = 10	Cerebral cortex	MUFAs (C16:1 and C24:1)↑		
			SFAs (C20:0 and C24:0)↑		
			PUFAs (C20:4, C20:2,C22:6)↓		
			SFA (C15:0)↓		
Human subjects	AD = 14	Post-mortem middle frontal gyrus, inferior frontal gyrus	Linoleic acid↓	LC–MS	[23]
	CH = 14		Linolenic acid↓		
	ASYMAD = 15		EPA↓		
			Oleic acid↓	GC–MS	
			Arachidonic acid↓		
			DHA↑		
		Post-mortem cerebellum	DHA↑		
Human subjects	AD = 114	Frontal cortex	Oleic acid↑	GC–MS	[24]
	CH = 58		Stearic acid↓		
		Post-mortem temporal cortex	Oleic acid↑		
			Stearic acid↓		
			Arachidonic acid↓		
		Post-mortem parietal cortex	PA↑		
Human subjects	AD = 12	Plasma	FFA↓	GC–MS	[25]
	MCI = 12		Oleic acid↓		
	CH = 12		PA↓		
			EPA↓		
			DHA↓		
		Post-mortem frontal cortex	DHA↓		
			Oleic acid↑		
		Post-mortem temporal cortex	DHA↓		
			Oleic acid↑		
Human subjects	AD = 30	Plasma	Arachidic↑	GC–MS	[26]
	MCI = 14		Erucic↑		
	CH = 30		Vaccenic acid ↑		
			Cerotic↓		
			Linoleic acid ↓		
Human subjects	AD = 103	Plasma	Vaccenic acid↑	GC-FID	[27]
	MCI = 92	CSF	Erucic acid↑		
	CH = 94		Oleic acid↓		
			DHA↓		
Human subjects	AD = 148	Plasma	PC↑	LC–MS	[10]
	CH = 152		TG–		
Human subjects	AD = 30	Post-mortem prefrontal cortex	DAG↑	LC–MS	[28]
	CH = 28		SP↑		
		Post-mortem entorhinal cortex	Lysobisphosphatidic acid↑		
			Sphingomyelin↑		
			Ganglioside GM3↑		
			Cholesterol esters↑		
Human subjects	AD = 34	Post-mortem gray matter	MAG↑	LC–MS/MS	[29]
	MCI = 19		DAG↑		
	CH = 28		PE↓		
			PL↓		
Human subjects	AD = 19	CSF	Lysophosphatidylcholine↓	GC–MS	[30]
	CH = 19				
Human subjects	AD = 20	Serum	PE↓	LC–MS/MS	[31]
	CH = 19		PL↓		
Human subjects	AD = 40	Serum	PL↓	LC–MS	[32]
	CH = 66				
Human participants and C57BL6 mice	AD = 24	Post-mortem gray matter White matter (frontal cortex)	PL↓	ESI/MS	[33]
	CH = 6				
	AD = 5	White matter (cerebellum)	PL↓		
	WT = 5	Cerebral cortex	PL↓		
J20 mice	AD = 3	Hippocampal region	Plasmalogen-ethanolamine (9 months)↑	LC-SRM/MS	[34]
	WT = 3		Plasmalogen-ethanolamine (15 months)↓		
Human subjects	AD = 6	Post-mortem cerebral cortex	SM (32:1), SM (32:2), SM (36:3), SM (38:3)↑	LC–MS/MS	[35]
	Cerad-b = 7		Cer↑		
	CH = 6		PS↑		
			Lysophosphatidylglycerol↑		
Human subjects	AD = 5	Post-mortem frontal cortex (Brodmann area 9)	PC (32:1), PC (34:0)↑	MALDI-MSI	[36]
	MCI = 5		Sphingomyelin (38:1)↑		
	CH = 5		S1P 1↓		
Human subjects	AD = 42	Plasma	PC 16:0/20:5, PC16:0/22:6, PC18:0/22:6↓	LC–MS	[37]
	MCI = 50				
	CH = 49				
Human subjects	AD = 15	Post-mortem neocortex	Cer↑	LC–MS/MS	[38]
	Mild AD = 15		DAG↑		
	CH = 16		Lysophosphatidylglycerol↑		
			TG↑		
			PE↑		
			Phosphatidylglycerol↑		
Human subjects	AD = 40	Plasma	Cer↑	LC–MS/MS	[39]
	CH = 42		SM↓		
			PC↑		
			PE↓		
			TG↑		
Human subjects	*n* = 22 with CDR from 0 to 3	Post-mortem white matter (frontal, parietal, cerebellum)	Cer↑	ESI/MS	[40]
Human subjects	*n* = 34 with varying Braak NFT staging	Post-mortem hippocampus, gray matter (temporal)	S1P↓	LC–MS/MS	[41]
Human subjects	AD = 21	CSF	S1P↑	UHPLC-TOF MS	[42]
	MCI = 33				
	CH = 21				
Human subjects	AD = 192, CH = 800	Plasma	Cer (male)↑	LC/ESI/MS/MS	[43]
			SM (female)↓		

↑: increase; ↓: decrease; -: no significant change; CH: cognitive health; MCI: mild cognitive impairment; AD: Alzheimer’s disease; CSF: cerebrospinal fluid; GC–MS: gas chromatography-mass spectrometry; LC–MS: liquid chromatography-mass spectrometry; GC-FID: gas chromatography with flame ionization detection; ESI/MS: electrospray ionization mass spectrometry UHPLC-TOF; MS: ultra-high performance liquid chromatography-time-of-flight mass spectrometry; FFA: free fatty acids; DHA:docosahexaenoic acid; NP: brain-derived nanoparticles; PA: palmitic acid; MUFA: monounsaturated fatty acid; PUFA: polyunsaturated fatty acid; SFA: saturated fatty acid; WT: wild-type mouse; ASYMAD: asymptomatic Alzheimer’s disease; EPA: eicos-apentaenoic acid; PC: phosphatidylcholine; TG: triglyceride; DAG: diacylglycerol; SP: sphingolipids; MAG: monoacylglycerol; PE: phosphatidylethanolamine; PL: plasmalogen; Cerad-b: classified as intermediate probability of AD; SM: sphingolipid; Cer: ceramide; PS: phosphatidylserine; MALDI-MSI: matrix-assisted laser desorption ionization as an imaging mass spectrometry method; S1P: sphingosine 1-phosphate

### Changes in lipid levels in AD

Several studies have reported that lipid levels vary across tissues under the influence of AD pathology. Some lipid alterations in the CNS parallel those in peripheral tissues. For instance, plasmalogen (PL) levels are significantly reduced in the serum, cerebral cortex, and hippocampus of AD patients [31,32]. The levels of monoacylglycerol and diacylglycerol are both elevated in the prefrontal cortex and gray matter of AD brains [28,29], and similar increases have been observed in the blood of AD model mice [44]. Linoleic acid (LA) levels are decreased in the middle frontal gyrus and inferior temporal gyrus of AD brains [23], with consistent reductions detected in the peripheral blood plasma [26]. Palmitic acid (PA) levels are significantly elevated in the neocortex (Brodmann area 7) of AD patients and in the cerebral cortex of APP/PS1 transgenic mice [21,22]. Elevated PA levels have also been observed in the serum of AD patients [45]. Notably, sex differences have been reported. For example, one study revealed that the increase in brain PA level was significantly greater in male AD patients than in female AD patients, although the underlying mechanisms remain unclear [21]. Similar sex-specific patterns have been reported for triglyceride (TG) and total cholesterol (TC) levels. A longitudinal study with a seven-year follow-up revealed that high TG levels are associated with increased AD risk in males, whereas no significant association was observed in females. Conversely, low TG levels are associated with reduced AD risk in females but not in males [46]. A cross-sectional study further indicated that elevated levels of TC and low-density lipoprotein cholesterol (LDL-C) may increase AD risk in female *APOE* ε4 carriers, but no significant association was found in males [47]. However, another long-term study with a 25-year follow-up showed no association between elevated cholesterol levels and AD risk in females [48]. The mechanisms underlying these inconsistent findings are not fully understood, but several hypotheses have been proposed. One possibility is that sex hormones play a critical role in regulating lipid metabolism. Estrogen, in particular, has been shown to exert neuroprotective effects [49]. The risk of AD increases sharply in postmenopausal females [50], suggesting that hormonal differences between sexes may partially explain the observed disparities. In addition, compared with males, females exhibit higher levels of peripheral inflammation [51], which is considered a central contributor to AD pathogenesis [52]. These differences in inflammatory profiles may also contribute to sex-specific lipid alterations associated with AD. Given these findings, future studies should be designed to assess sex-specific effects through recruitment of balanced numbers of male and female participants, stratification by sex, and reporting sex-disaggregated results. Furthermore, potential contributors to sex differences—such as other sex hormones (e.g., progesterone and androgens), sex-specific brain structures or functions, and genetic variations—should be examined to better elucidate the role of dysregulated lipid homeostasis in AD risk in males and females.

However, some lipids exhibit disparate changes between the CNS and peripheral tissues. For example, oleic acid (OA) levels are elevated in the frontal and temporal cortices of the brain [24] but reduced in plasma of AD patients [25]. Phosphatidylcholine (PC) levels are significantly increased in the cerebral cortex, prefrontal cortex, and cerebrospinal fluid (CSF) of AD patients [35,36,53] but significantly decreased in peripheral blood [37,54]. In contrast, phosphatidylethanolamine (PE) and phosphatidylserine (PS) levels are significantly decreased in the brains of AD patients [55], but no significant changes have been detected in circulating plasma [39]. Most studies have reported reduced levels of docosahexaenoic acid (DHA), a dietary omega-3 fatty acid, in the brains of AD patients, particularly in the hippocampus, a region closely associated with AD pathology [25,56]. However, some studies have reported elevated DHA levels in the middle frontal and inferior temporal gyri of AD patients [23], whereas others have reported decreased serum DHA levels [57]. These findings suggest not only disparities between central and peripheral DHA concentrations but also regional heterogeneity in the distribution of DHA within the AD brain. Consistently, sphingosine-1-phosphate (S1P) levels have been shown to be reduced by 66% in the hippocampus and 64% in the inferior temporal gyrus of AD patients, with no significant differences observed in the superior frontal gyrus or cerebellum [41]. The regional specificity of S1P levels may be attributed to the differential activity of sphingosine kinases 1 and 2 across brain regions. Specifically, reduced activity of sphingosine kinases 1 and 2 in the hippocampus and inferior temporal gyrus may contribute to the significantly lower S1P levels observed in these regions [58]. Sphingomyelin (SM) levels are significantly decreased in the plasma of AD patients and exhibit region-specific alterations in the brain. Most studies have reported elevated SM levels in the gray matter, CSF, and cerebellum of AD patients [59–61], whereas some have reported decreased SM levels in the frontal mesolimbic region [62]. However, the mechanisms underlying these region-specific changes remain poorly understood. As has been proposed for S1P, the differences in SM levels may be influenced by the regionally heterogeneous activity of lipid-metabolizing enzymes. Further research is needed to elucidate the roles of these enzymes in specific brain regions, which may help clarify the mechanisms driving the region-specific lipid dysregulation in AD. The levels of ceramide (Cer), a central bioactive sphingolipid, have consistently been found to be elevated in both the central and the peripheral compartments in AD patients, including the CSF, cerebral cortex, and plasma [39,63,64]. These increases are thought to stem, at least in part, from SM hydrolysis [40,63], which potentially accounts for the observed reductions of SM level in the context of AD. However, Cer, SM, and S1P are metabolically interconnected: SM can be hydrolyzed into Cer, and Cer can be reconverted into SM. Despite this relationship, the causal associations underlying the interconversion of these molecules in the context of AD remain unclear [65]. Cer can also be synthesized via several distinct biosynthetic pathways, including the de novo pathway, the salvage pathway, and the sphingomyelinase pathway [66]. Among these pathways, the salvage pathway has been most notably implicated in AD pathology [67,68]. Thus, attributing the increased Cer levels solely to SM hydrolysis may oversimplify the underlying mechanisms. Although the existence of multiple Cer synthesis pathways is well established, the mechanisms through which Cer produced via different biosynthetic routes contributes to AD pathophysiology remains incompletely understood [68]. Future studies should be conducted to investigate whether Cer derived via specific pathways exerts differential effects on AD progression and elucidate the molecular mechanisms involved. Furthermore, research should explore sphingolipid interconversion across different stages of AD to deepen our understanding of the dynamic and region-specific roles of sphingolipids in disease development.

### Effects of lipids on Aβ levels

Excessive deposition of Aβ is a key pathological mechanism through which lipids influence the progression of AD. Consumption of a high-PA diet for 8 weeks significantly increased Aβ production and tau protein phosphorylation in the hippocampus and cortex of C57BL/6 mice. During this process, PA activates G protein-coupled receptor 40 (GPR40) in SK-N-MC cells, which subsequently upregulates the phosphoinositide 3-kinase (PI3K)/Akt signaling pathway. This activation mediates increased expression of amyloid precursor protein (APP) and beta-site APP-cleaving enzyme 1 (BACE1). In addition, PA stimulates both Akt/mTOR/HIF-1α and Akt/NF-κB (nuclear factor kappa-light-chain-enhancer of activated B cells) signaling, further increasing APP and BACE1 expression in SK-N-MC cells and amplifying Aβ production [69]. In a 3 × Tg-AD mouse model, consumption of a high-PA diet for 3 months impaires the endogenous antioxidant defense system in the hippocampus and increases glutathione depletion, triggering oxidative stress. The increased oxidative stress is closely associated with NF-κB activation, which in turn increases the expression and enzymatic activity of BACE1, ultimately promoting Aβ accumulation [70].

Cer has also been shown to promote Aβ formation by regulating APP cleavage [71]. C6-Cer, a short-chain Cer, was similarly found to increase APP cleavage by BACE1, leading to increased Aβ biosynthesis [66]. Animal studies further demonstrated that Cer contributes to Aβ accumulation in vivo by affecting exosome dynamics. Increased production of Cer-containing exosomes is associated with increased Aβ levels. Inhibition of exosome formation and secretion in the brains of AD mice using neutral sphingomyelinase-2 attenuated Aβ deposition and tau hyperphosphorylation, ultimately ameliorating AD-related pathology and improving cognitive performance [72,73].

Dysregulation of SM homeostasis also contributes to AD pathology. Disruption of the SM balance increases Aβ oligomerization within SM-rich lipid bilayers, promoting the formation of Aβ oligomers and increasing AD risk [74]. Disruption of SM homeostasis also promotes BACE1 activity in the hippocampus of APP/PS1 mice, leading to increased Aβ production [75].

High cholesterol levels have also been shown to increase the BACE1 proteolytic activity and the exposure of the β-cleavage site on APP, thereby promoting Aβ production through the amyloidogenic pathway [76,77]. Furthermore, cholesterol may inhibit the nonamyloidogenic pathway by binding to specific sites on α-secretase, reducing its activity and contributing to Aβ accumulation [78].

In contrast, three months of DHA treatment reduced Aβ production in Tg2576 mice by limiting the APP secretase processing. This effect was achieved through regulation of membrane order and fluidity, reduction of oxidative stress, and inhibition of the PI3K and glycogen synthase kinase 3α (GSK3α) signaling pathways. Similarly, in an AD rat model, 12 weeks of DHA supplementation reduced Aβ accumulation in the brain by upregulating the expression of insulin-degrading enzyme (IDE), thereby increasing Aβ clearance [79,80]. In vitro studies further demonstrated that linoleic acid accumulation in neuronal membranes inhibits the neurotoxic production of Aβ in neurons [81]. Consistent with this finding, in AD model mice fed with an OA-enriched diet, an increased Aβ_40_/Aβ_42_ ratio was observed, accompanied by decreased levels of BACE1 and progerin as well as reduced formation of Aβ plaques [82]. An imbalance in the lateral homeostasis of phosphatidylcholines (PCs), key structural components of cell membranes, impairs membrane functionality by reducing the efficiency of Ca^2^⁺-dependent vesicle fusion, which in turn diminishes membrane fluidity and elasticity [83]. These changes negatively affect synaptic transmission and APP processing, ultimately leading to increased Aβ production [84]. Moreover, a clinical trial demonstrated that 180-day oral administration of the PC derivative choline alfoscerate improved cognitive and dementia-related behavioral symptoms in patients with mild to moderate AD. This effect appeared to result from an increase in acetylcholine levels in the hippocampus, which enhanced cholinergic neurotransmission and activated the brain-derived neurotrophic factor (BDNF) survival pathway, thereby reducing Aβ deposition [85,86]. PS also plays an important role in Aβ clearance. In vitro studies using Neuro2a cells demonstrated that the neuron-derived exosomes promote uptake of Aβ fibrils by microglia in a PS-dependent manner, thereby promoting Aβ degradation and clearance [72].

The specific roles of S1P in the pathogenesis of AD remain inconclusive. On the one hand, several studies have suggested that S1P exerts a protective effect against AD. For instance, S1P levels in the cerebral cortex are significantly negatively associated with Aβ deposition in the brain [58]. On the other hand, some evidence indicates that S1P may contribute to AD progression. S1P has been shown to directly increase the neuronal BACE1 activity in the brains of AD mice, thereby promoting Aβ production. Furthermore, aberrant S1P/S1PR (sphingosine-1-phosphate receptor) signaling in the cerebral cortex upregulates the Akt/mTOR/tau pathway in an AD mouse model, ultimately resulting in excessive Aβ accumulation and tau hyperphosphorylation [87,88].

Notably, a reciprocal relationship exists between Aβ and lipid membranes. Alterations in lipid levels, as previously described, influence the production and toxicity of Aβ, and Aβ in turn affects lipid membranes [89]. Three models of Aβ-mediated membrane damage have been proposed: (1) generation of stable transmembrane protein pores, (2) membrane destabilization via a “carpet model”, and (3) removal of lipid components from the bilayer via a detergent-like mechanism [90]. Specifically, interactions between early-stage Aβ and the membrane can modulate lipid dynamics (such as leading to more rapid lipid headgroup motion and reduced lateral diffusive motion), and ultimately influence the membrane structural interfaces and Aβ fibrillation [91,92]. A recent cryo-electron microscopy study further revealed that during Aβ_40_ aggregation, lipids interact with Aβ_40_ to form lipid micelles on the fibril surface. This study provided molecular-level insights into how Aβ aggregation leads to lipid extraction from vesicles, a process that plays a key role in elucidating the AD pathology [93]. Furthermore, the effects of Aβ on membranes may be influenced by the lipid composition and brain region. For example, Aβ increases neuronal membrane fluidity in the cerebral cortex and hippocampus, whereas no significant effect is observed in the cerebellum. Cholesterol-rich neuronal membranes may promote Aβ accumulation via hydrophobic interactions [94]. Additionally, Ca^2^⁺ dyshomeostasis may be a key mediator of Aβ-induced membrane damage. Aβ oligomers facilitate excessive Ca^2^⁺ influx either by forming Ca^2^⁺-permeable channels or by disrupting the lipid architecture. Ca^2^⁺ overload further disrupts the lipid bilayer structure and promotes oxidative reactions, thereby exacerbating Aβ toxicity [95]. Conversely, inhibition of Ca^2^⁺ dysregulation can reduce membrane damage and attenuate Aβ-associated cytotoxicity [96,97]. In summary, the interaction between Aβ and lipid membranes has emerged as an important research direction for elucidating the pathological mechanisms of AD. Although substantial and exciting progress has been made, further investigations are still needed—particularly studies using nanoscale imaging techniques to resolve the dynamic conformational changes of Aβ at the membrane surface and to clarify the causal relationships between specific lipid components or abnormal lipid levels in distinct brain regions and the toxicity of different types of amyloid assemblies. Such studies are expected to provide a more comprehensive understanding of Aβ–membrane interactions in AD and to offer a theoretical basis for potential therapeutic strategies targeting lipid homeostasis or membrane integrity.

### Other effects of lipids correlated with AD

Lipids contribute to the pathogenesis of AD through several other pathways in addition to the Aβ-related mechanisms. For instance, tau protein, like Aβ, can interact with lipid membranes [98]. The two-dimensional nature of membranes can allow the concentration of tau on the surface, promoting the formation of tau oligomers and fibrils. Moreover, the presence of anionic lipids in membranes facilitates favorable electrostatic interactions with positively charged residues in tau, further enhancing its aggregation [99,100]. However, tau can also bind to membrane interfaces to form protein–lipid complexes, partially solubilizing the lipid bilayer and disrupting membrane integrity. This not only causes local membrane damage but also may promote the transcellular spread of tau pathology [101,102].

Furthermore, PA exacerbates tau protein phosphorylation at serine residues Ser199/202 and Ser214 by activating the glycogen synthase kinase-3β (GSK3β), leading to excessive accumulation of phosphorylated tau in neurons [103]. Elevated PA levels also induce lipotoxicity. In rat cortical astrocytes, excessive PA promotes the production of reactive oxygen species (ROS) and causes mitochondrial membrane potential collapse, ultimately resulting in astrocyte apoptosis, a process closely associated with AD progression [104]. Moreover, exposure of nerve growth factor-differentiated PC12 cells and rat cortical cells to high concentrations of PA caused upregulation of expression of proapoptotic genes encoding BNIP3 and the Fas receptor and increased oxidative stress. These changes contributed to mitochondrial depolarization and further ROS accumulation [105]. Notably, PA has also been reported to significantly reduce the expression of glucose transporter 1 in astrocytes, thereby inhibiting glucose uptake and lactate release. These metabolic abnormalities are hallmarks of AD [106,107]. In addition to affecting astrocytes, PA also exacerbates AD pathology by inducing inflammatory responses in microglia [108].

Cer also contributes to AD progression by regulating apoptosis. Cer can activate both caspase-dependent and caspase-independent apoptotic pathways, thereby promoting neuronal cell death [109]. Furthermore, Cer increases ROS generation and reduces neuronal viability by inhibiting the PI3K/Akt signaling pathway. It also increases proapoptotic activity by downregulating the expression of the anti-apoptotic protein Bcl-2 and increasing the mRNA and protein levels of the proapoptotic proteins Bax and Hrk [110].

Cholesterol accumulation is associated with cognitive impairment and hippocampal atrophy, primarily through its proapoptotic effects on hippocampal neurons [111]. Additionally, cholesterol disrupts the clearance of microtubule-associated protein tau and Aβ by impairing autophagic processes. Specifically, cholesterol enrichment in the endosomal–lysosomal membranes inhibits autophagosome–lysosome fusion, leading to the accumulation of autophagic vesicles containing microtubule-associated protein tau and Aβ and worsening of Aβ-induced mitochondrial dysfunction [112]. In astrocytes, cholesterol accumulation alters the secretion of exosomes carrying Aβ-related peptides; the uptake of these exosomes by neurons induces neurotoxicity [113]. In microglia, 25-hydroxycholesterol promotes neuroinflammation in an APOE isoform-dependent manner [114]. Furthermore, dysregulation of lipid metabolism impairs oligodendrocyte function, thereby reducing myelin production, contributing to white matter abnormalities, and ultimately exacerbating AD pathology [115]. It is important to emphasize that due to the presence of the blood–brain barrier (BBB), cholesterol in the CNS is synthesized in situ, and the impact of peripheral cholesterol on the CNS homeostasis is minimal [116].

DHA has been shown to inhibit tau phosphorylation at Ser422 and Ser396 by suppressing the activity of c-Jun N-terminal kinase (JNK), thereby improving learning and motor performance in mice [117]. In addition, consumption of a medium-chain triglyceride (MCT) diet significantly reduced both tau phosphorylation and Aβ levels in the cortex and hippocampus of mice. The underlying mechanism may involve the inhibition of proinflammatory factors, including tumor necrosis factor-α (TNF-α) and NF-κB, leading to attenuated neuroinflammation. The MCT diet also promoted the expression of BDNF and reduced the glial fibrillary acidic protein (GFAP) level, potentially exerting a neuroprotective effect [118].

Alpha-linolenic acid (ALA) has also demonstrated neuroprotective effects. ALA inhibits the activation of toll-like receptor 4 and its downstream effectors, including phosphorylated JNK, phosphorylated NF-κB p65, and TNF-α, in mice subjected to intracerebroventricular injection of Aβ. ALA treatment significantly reduces GFAP expression and levels of proapoptotic markers, ultimately protecting against neuronal cell death and neurodegeneration [119].

In addition to ALA, cis-9, trans-11-conjugated linoleic acid (c9,t11-CLA) has shown beneficial effects in AD models. Administration of c9,t11-CLA for seven weeks or longer reduced hippocampal Aβ levels in AD model mice. This decrease was accompanied by an increase in the number of astrocytes secreting anti-inflammatory cytokines, such as interleukin (IL)-10 and IL-19, and a decrease in proinflammatory cytokine levels in the cerebral cortex, thereby attenuating the inflammatory response [120,121]. Inhibition of arachidonic acid (ARA) metabolism has also been reported to reduce oxidative stress and inflammation in microglia, which may help delay AD progression [122]. PL has been shown to alleviate Aβ-induced toxicity in neural stem cells. Mechanistically, PL regulates neural stem cell differentiation by activating the Wnt/β-catenin signaling pathway, thereby enhancing adult hippocampal neurogenesis and improving learning and memory functions in AD mice [123]. Exogenous S1P can activate S1P receptor subtypes 1 and 3 in hippocampal neurons. This activation downregulates the expression of the proapoptotic protein Bcl-2 and inhibits neuronal apoptosis, thereby increasing neuronal viability and activity and ultimately mitigating the pathological progression of AD [124].

### Dysregulation of lipid homeostasis is an upstream core event in AD

It is well known that imbalances in lipid homeostasis in both the brain and the blood contribute substantially to the onset and progression of AD, primarily through disruption of lipid metabolism, alterations of neuronal membrane structure, and synaptic dysfunction [19]. Studies have reported abnormal changes in several lipid species—including phosphatidylcholine, sphingolipids, and cholesterol—in the CSF of cognitively normal individuals and individuals at the very early stages of AD [125]. In animal models, high-fat diets induce dysregulation of lipid homeostasis, accompanied by excessive deposition of Aβ and elevated tau phosphorylation in brain tissue—features characteristic of AD pathology [69]. These findings suggest that disruptions in lipid metabolism may be an upstream event in the pathogenesis of AD. Large-scale epidemiological studies have shown that plasma cholesterol levels are significantly elevated during middle age, a pattern also observed in individuals with AD [126]. Conversely, other studies have reported significantly lower cholesterol levels in older adults than in normal younger adults [127]. Moreover, moderate cholesterol supplementation has been shown to improve synaptic function, long-term depression, and learning and memory performance in rats [128]. These findings indicate that both elevated and decreased cholesterol levels are reflective of lipid metabolism abnormalities, underscoring the broader role of lipid dysregulation in AD development. In addition, in vitro studies have demonstrated that dysregulation of lipid homeostasis can result in the activation of microglia and astrocytes, thereby promoting neuroinflammation and inducing abnormal phosphorylation of tau proteins involved in Aβ production [129]. In animal models, consumption of high-cholesterol diets exacerbates insulin resistance, reduces cortical acetylcholine levels, enhances neuroinflammatory responses, and upregulates APP expression. Collectively, these changes contribute to Aβ overproduction, increased tau phosphorylation in the brain, and eventually cognitive impairment [130,131]. Notably, long-term use of statins, which regulate lipid metabolism, has been associated with a significantly reduced risk of developing AD [132,133]. Together, these findings suggest that the dysregulation of lipid homeostasis may be a key upstream factor in the initiation and progression of AD.

**Fig. 1:**
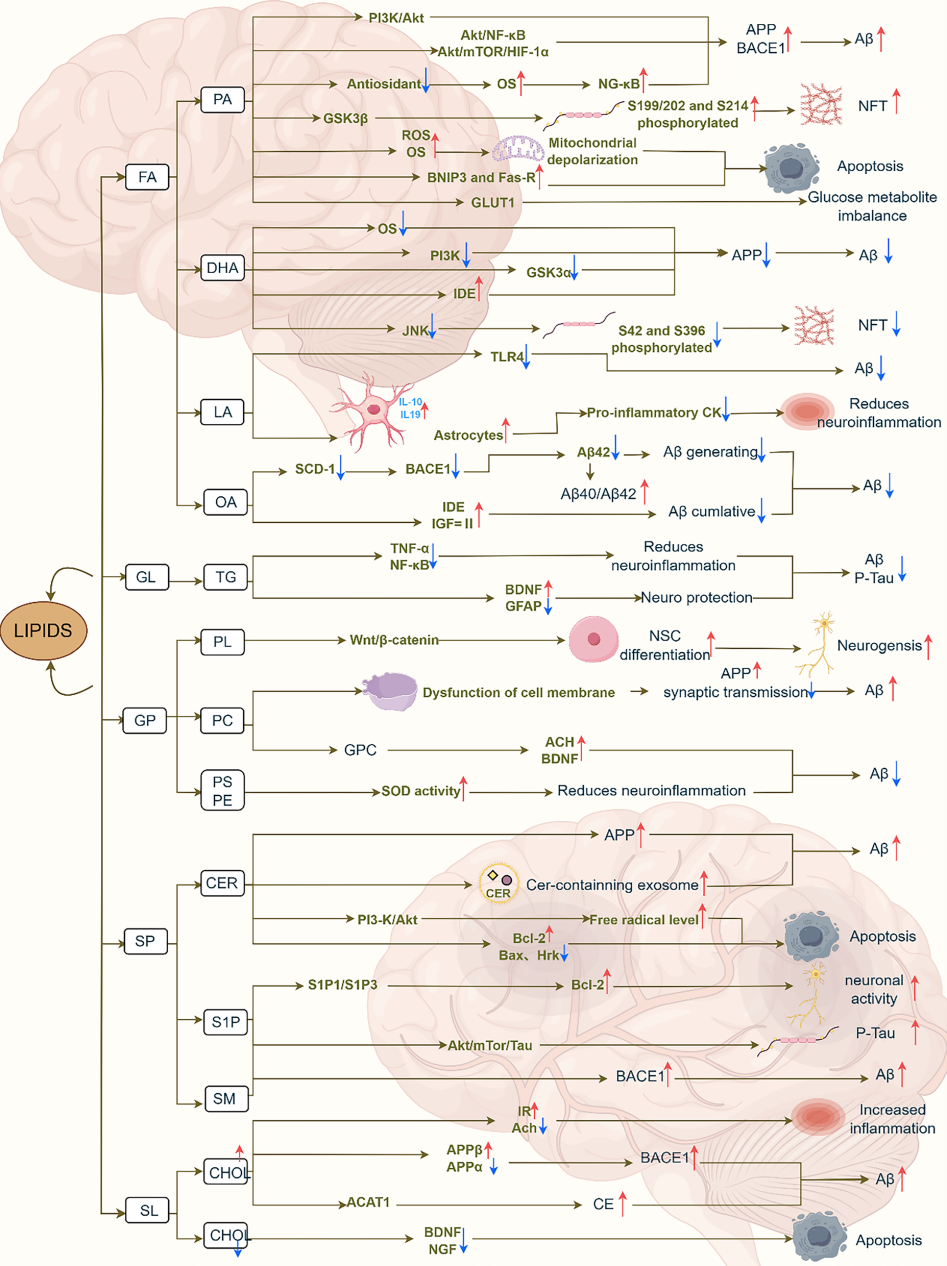
Molecular mechanisms by which multiple lipids influence AD. Note : FA: fatty acid; PA: palmitic acid; APP: amyloid precursor protein; BACE1: beta-site amyloid precursor protein cleaving enzyme 1; Aβ: amyloid-β; OS: oxidative stress; NFT: neurofibrillary tangle; ROS: reactive oxygen species; GLUT1: glucose transporter 1; DHA: docosahexaenoic acid; IDE: insulin-degrading enzyme; JNK: c-Jun N-terminal kinase; LA: linoleic acid; TLR4: toll-like receptor 4; OA: oleic acid; IL-9: interleukin-9; IL-10: interleukin-10; SCD-1: Stearoyl-CoA desaturase-1; IGF: insulin-like growth factor; GL: glycerolipids; TG: triglyceride; TNF-α: tumor necrosis factor-α; BDNF: brain-derived neurotrophic factor; GFAP: glial fibrillary acidic protein; GP: glycerophospholipids; PL: plasmalogen; NSC: neural stem cell; PC: phosphatidylcholine; ACH: acetylcholine; PS: phosphatidylserine; PE: phosphatidylethanolamine; SOD: super oxide dismutase; SP: sphingolipids; CER: ceramide; Bcl-2: anti-apoptotic-2; S1P: sphingosine-1-phosphate; SM: sphingomyelin; CHOL: cholesterol; IR: insulin resistance; CE: cholesteryl ester; NGF: nerve growth factor.

With continuing advances of research techniques, particularly the application of genome-wide association studies, genetic variants involved in lipid metabolism that may influence lipid levels and, in turn, affect the risk of AD, have been identified. Genes such as *APOE*, ATP-binding cassette subfamily A member 1 (*ABCA1*), *ABCA7*, and sterol regulatory element-binding protein 2 (*SREBP2*) have been shown to be closely associated with AD pathology [134]. Lipidomics enables a comprehensive comparison of lipid profiles between AD patients and healthy controls, and facilitates the longitudinal tracking of lipid alterations during the progression of AD as well as the evaluation of potential therapeutic targets. Mass spectrometry further increases the utility of this analysis by accurately determining the types and concentrations of lipids in biological specimens, allowing the precise quantification and spatial mapping of lipid changes in the context of AD. Lipidomics and mass spectrometry have been utilized in several studies to explore the relationship between lipid alterations and AD, with the findings underscoring the important role of lipid metabolism in AD pathology [10,135,136]. Additionally, machine learning methods are well suited for handling large, complex datasets and have been employed to integrate results from genome-wide association studies, mass spectrometry analyses, and lipidomic analyses in an efficient, consistent, and accurate manner to construct predictive models. These models have been used for the diagnosis and prediction of AD, with applications such as biomarker analyses of CSF, plasma, and other biological sources [137,138]. The development and application of these emerging technologies provide promising opportunities for advancing targeted AD therapies and for paving the way toward personalized and precision medicine. The specific molecular mechanisms through which various lipid species may influence AD pathogenesis are shown in Fig. 1. Notably, when the role of a specific lipid in AD is investigated, tissue specificity should be carefully considered. Moreover, the broader regulatory network of lipid metabolism must be considered, reflecting the inherently complex nature of lipid biology.

## Lipid-targeting therapies for AD

### Pharmacological therapy

Current strategies for management of AD primarily involve behavioral/lifestyle modifications and pharmacological interventions. To date, multiple drugs have been used for AD prevention and treatment, including acetylcholinesterase inhibitors (e.g., donepezil, rivastigmine, and galantamine), NMDA receptor antagonists (e.g., memantine), and monoclonal antibodies targeting Aβ (e.g., lecanemab and donanemab). These drugs can partially alleviate AD symptoms or slow disease progression [142,143]. In recent years, drugs targeting lipid dysregulation in AD have been extensively investigated. Lipid-targeting AD therapeutics function primarily by activating lipid-sensitive nuclear receptors or reducing lipid accumulation.

The retinoid X receptor (RXR), liver X receptor (LXR), and peroxisome proliferator-activated receptor (PPAR) belong to the nuclear receptor superfamily. By activating genes involved in lipid metabolism, these receptors serve as key regulators of peripheral and central lipid homeostasis [144]. Several strategies targeting these receptors have been explored in AD. For instance, bexarotene, an RXR agonist, increased Aβ clearance and improved cognitive deficits in AD mouse models by upregulating ABCA1 activity and reversing APOE4 hypolipidation [145]. However, clinical studies showed that it did not significantly affect Aβ metabolism or cognitive function [146,147]. PPARγ agonists mitigated Aβ pathology and improved cognitive function by modulating Aβ production and clearance in animal models [148,149]. In human trials, although the PPARγ agonist pioglitazone showed promising preliminary results [150,151], a recent large-scale phase III trial in high-risk AD patients revealed that pioglitazone did not delay the onset of mild cognitive impairment [152]. Similar outcomes were observed in clinical evaluations of another thiazolidinedione PPARγ agonist, rosiglitazone [153]. Notably, in a phase II trial in patients with mild-to-moderate AD, rosiglitazone selectively improved cognition in *APOE4*-noncarriers at the higher doses tested, but showed no efficacy in *APOE4*-carriers [152].

With respect to reducing lipid accumulation, statins inhibit HMG-CoA reductase (HMGR) to suppress the synthesis of sterols (e.g., cholesterol) [154]. Ex vivo and animal model studies have demonstrated that statins strongly reduce Aβ levels [155,156]. In human studies, statins have been shown to reduce the risk of dementia and AD in patients with a normal cognitive status at baseline [157]. However, a Cochrane systematic review incorporating two randomized controlled trial (RCT) involving 26,340 participants concluded that statins had no effect on the dementia occurrence rate or cognitive decline [158]. Thus, the outcomes of clinical trials for lipid-targeting AD therapies remain highly controversial. However, as accumulating evidence supports the pivotal role of lipid metabolism in AD pathophysiology, there is a need to elucidate the precise mechanisms of lipid regulation and the interactions of lipids with pathological markers such as Aβ and tau. Furthermore, integrating personalized treatment strategies—such as drug selection on the basis of *APOE* genotype—may increase the clinical efficacy of lipid-targeting therapies.

### Exercise therapy

Exercise is an effective nonpharmacological intervention for the prevention and treatment of AD. Both human and animal studies have demonstrated that regular exercise improves neuropathological features and cognitive function in AD patients and AD model mice [159,160]. Specifically, regular exercise regulates lipid metabolism [161,162], activates neurotrophic factors [163], reduces Aβ aggregation [164], decreases NFT formation [165], suppresses inflammatory responses [166], alleviates oxidative stress [167], and enhances neurovascular unit function [168]. Furthermore, regular exercise plays a crucial role in maintaining and restoring systemic lipid homeostasis, thereby showing considerable potential for mitigating AD through modulation of lipid homeostasis. As discussed earlier, the lipid system is vast and complex, with levels of its components influenced by multiple factors, including lipid distribution, AD progression stage, and age. Given the complexity of the lipid system and the controversies surrounding lipid-targeting pharmacological therapies, exercise—as a noninvasive, multiorgan and multitarget holistic intervention—demonstrates unique advantages in regulating systemic lipid homeostasis. Exercise modulates the levels of various lipid molecules, thereby achieving effective overall regulation of lipid homeostasis. In the following, we will summarize the molecular mechanisms through which exercise influences AD through regulation of lipid homeostasis, and discuss the peripheral and central crosstalks between exercise-induced lipid modulation and AD, as well as the impact of different *APOE* genotypes on the effects of exercise in AD. Table 2 summarizes the effects of different exercise interventions on lipid levels across various tissues.

**Table 2:** Effects of different exercise interventions on the levels of multiple lipids in various tissues

Subjects	Groups	Exercise type	Lipid changes	Sample/tissue	References
Human subjects	*n* = 6	Resting for 30 min and cycling 4 h 30%VO_2MAX_	FFA↑	Blood	[169]
Human subjects	EX = 10, CON = 10	CON: rehabilitation therapy for 30 min each day	TG↓	Blood	[170]
		EX: Rehabilitation therapy for 30 min each day, plus cycling at intensity of 50%-70%THR for 5 times a week, 30 min each time, for 12 weeks	FFA↓		
			HDLC↑		
Sprague–Dawley rats	SED = 10, MICT = 8, HIIT = 6	MICT: treadmill	PA↓	Serum	[171]
		1-min 35%-40% VO_2MAX_ 45-min 75%-80% VO_2MAX_			
		HIIT: 9 groups			
		4-min 45%-55% VO_2MAX_	Stearic acid↓		
		1-min 90%-95% VO_2MAX_	Octadecadienoic acid↓		
		8 months			
C57BL/6 mice	SED = 16	EX: Voluntary wheel running	PA↓	Cerebral cortex	[172]
	EX = 15	4 weeks	AA↑		
			DHA↑		
			PLA_2_↑		
Human subjects	CON = 12	EX: treadmill	CER↓	Plasma	[173]
	EX = 11	1–2 weeks	Sphingosine 1-phosphate↓		
		50%-60% HRR	Sphingomyelin↓		
		3–6 weeks	Sphinganine↓		
		60%-70%HRR	PE↓		
		3 times a week	PC↓		
		40 min each time	DHA↑		
		7–26 weeks	AA↑		
		70%-80% HRR			
		3 times a week			
		50 min each time			
Sprague–Dawley rats	SED = 18	EX: treadmill	TG↑	Gastrocnemius muscle,	[174]
	EX = 20	1–8 weeks		flounder muscle	
		20 m/min rise to 35 m/min			
		20 min rise to 1 h			
		8–20 weeks			
		20 m/min			
		10% gradient			
		15 min			
		28 m/min			
		10% gradient			
		30 min			
		35 m/min			
		10% gradient			
		15 min			
		3 times a week			
		20 weeks			
APP/PS1 double-transgenic mice and C57BL6 mice	CON = 10	EX1: treadmill	TC↓	Plasma	[15]
	EX1 = 10	45%-55% VO_2MAX_	TG↓		
	EX2 = 10	EX2: treadmill	LDLC↓		
	WTC = 10	60%-70% VO_2MAX_	HDLC↑		
		5 times a week			
		30 min each time			
		5 months			
Human subjects	CON = 82	EX: 70% mHR aerobics	HDLC↑	Plasma	[16]
	EX = 90	3 times a week			
		60 min each time			
		16 weeks			
C57Bl/6NCrl mice	CON = 12	EX: treadmill	PC↑	Liver	[175]
	EX = 25	warm-up: 5 m/min 5 min	PE↑		
		formal training: 13 m/min	PL↑		
		13% gradient			
		60 min			
Wistar rats	CON = 8	CON: treadmill	PL↑	Principal artery	[176]
	EX = 8	15 m/min for 5 min	CER↑		
		once a week	TC↓		
		EX: treadmill	CER↑	Cerebellum	
		25 m/min for 45 min in 1–12 weeks			
		25 m/min for 60 min in 12–24 weeks			
		5 times a week			
		24 weeks			
Human subjects	*n* = 44	EX: power cycling	PC↑	Muscle	[177]
		50% VO_2MAX_ 1.5 h	PE↑		
C57BL/6 J mice	WTC = 6	EX: treadmill	PC↑	Brain	[178]
	CON = 6	12 m/min for 1 h			
	EX = 6	3 times a week			
		2 weeks			
C57BL/6 mice	CON = 10	EX & EXP: treadmill	PA↓	Liver	[179]
	EX = 10	13 m/min for 40 min	PC↑		
	EXP = 10	5 times a week			
		18 weeks			
APP/PS1 double-transgenic mice	WTC = 6	EX: treadmill	PA↓	Intestine	[180]
	CON = 6	10 m/min for 5 min	PS↑		
	EX = 6	15 m/min for 20 min	PE↑		
		10 m/min for 5 min			
		5 times a week			
		20 weeks			
Human subjects	*n* = 44	EX: power cycling	CER↑	Serum	[181]
		50% VO_2MAX_ 1.5 h			
Human subjects	CON = 49	EX: HIIT	SP↓	Serum	[182]
	EX = 49	3 times a week			
		45 min each time			
		8 weeks			
Human subjects	*n* = 24	Treadmill	CER↓	Plasma	[183]
		1–4 weeks			
		50% VO_2MAX_			
		5–12 weeks			
		80% VO_2MAX_			
		5 times a week			
		1 h each time			
		12 weeks			
Wistar rats	CON = 10	EX1: treadmill	CER↓	Myocardium	[184]
	EX1 = 10	1200 m/h	PA↓		
	EX2 = 10	10% gradient for 3 h	Oleic↓		
		EX2: treadmill	Eicosapentaenoic↓		
		week 1, 960 m/h			
		week 2, 1200 m/h			
		week 3, 1440 m/h			
		weeks 4–5, 1680 m/h			
		7 times a week			
		40 min each time			
		5 weeks			
Human subjects	*n* = 15	EX: cycling	S1P↑	Plasma	[185]
Human subjects	CON = 10	EX: power cycling	S1P↑	Plasma	[186]
	EX = 10	VO_2MAX_ test			
		power cycling			
		70% VO2MAX for 1 h			
		CON: power cycling			
		70% VO_2MAX_ for 30 min			
Wistar rats	CON = 5	EX: treadmill	CER↓	Muscle	[187]
	EX = 5	1200 m/h	PA↓		
		10% gradient			
Human subjects	CON = 36	EX: high intensity exercise	PC↑	Plasma	[188]
	EX = 25		PE↑		
NSE/PS2m-transgenic mice	CON = 6	EX: treadmill	TC↓	Blood	[189]
	EX = 6	22 cm/s	TG↓		
		40 min each time	LDLC↓		
		3 months	HDLC↑		
NSE/APPsw Tg mice		EX: treadmill	TC↓	Serum	[190]
		16 weeks			

↑: increase; ↓: decrease; EX: exercise group; CON: control group; WTC: wild-type control group; THR: target heart rate; HRR: heart rate reserve; HDLC: High-density lipoprotein cholesterol; LDLC: low-density lipoprotein cholesterol; SED: sedentary group; MICT: moderate-intensity continuous training; HIIT: high-intensity interval training; FFA: free fatty acid; TG: triglyceride; AA: arachidonic acid; DHA: docosahexaenoic acid; PLA_2_: phospholipase A2; CER: ceramide; PC: phosphatidylcholine; PE: phosphatidylethanolamine; TC: total cholesterol; mHR: maximal heart rate; TG: triglyceride

#### Regulatory roles of exercise in lipid homeostasis

Studies have demonstrated that a single bout of vigorous exercise significantly reduced plasma PA levels in healthy males [191]. Both two-week high-intensity interval training (HIIT) and moderate-intensity continuous training were found to decrease serum PA levels in aged mice [171,191]. A similar phenomenon was observed in the cerebral cortex of young mice subjected to one month of voluntary wheel running [172]. A 26-week moderate-intensity aerobic exercise regimen significantly increased the blood levels of ARA, docosapentaenoate, DHA, and dihomolinoleate in patients with AD [173]. Both acute endurance and resistance training sessions significantly elevated plasma ARA levels in sedentary adults [192]. An 8-week combined aerobic and strength exercise program markedly increased plasma ARA levels in obese women [193]. Four weeks of voluntary wheel running significantly increased ARA levels in the cerebral cortex of healthy C57BL/6 J mice [172]. In addition, PL levels in the liver, aorta, brain, cerebellum, and hippocampus were increased by 24 weeks of treadmill exercise and 12 sessions of water maze training in healthy rats and AD model mice, respectively [176,194]. A single bout of acute exercise significantly increased the skeletal muscle PC content [177]. Similarly, a two-week aerobic exercise intervention modulated the abnormally elevated PC levels in the brains of methamphetamine-addicted mice [178]. Twenty weeks of aerobic exercise significantly increased the PE levels in the intestinal contents of APP/PS1 mice [180]. Treadmill exercise for 20 weeks elevated the PS levels in the intestinal contents of APP/PS1 mice [180], whereas 13 weeks of treadmill exercise increased the PS levels in the skeletal muscle of Wistar rats [195]. A single session of acute exercise significantly increased serum Cer levels during the exercise phase in diabetic patients, but these levels decreased below baseline during the recovery phase [181]. Eight weeks of HIIT reduced the circulating sphingolipid levels in middle-aged adults with high cardiometabolic risk [182]. Twelve weeks of supervised aerobic exercise decreased the plasma Cer levels in obese individuals and type 2 diabetes (T2D) patients [183], and a 16-week aerobic exercise intervention reduced the intramyocellular Cer levels in overweight/obese individuals [196]. A longer-term six-month aerobic exercise program decreased plasma Cer levels in T2D patients, accompanied by improvements in visuospatial memory [197]. Animal studies yielded consistent findings: six weeks of treadmill exercise reduced the total myocardial Cer content by 64% in Wistar rats [184] and decreased the Cer levels in the soleus and gastrocnemius muscles [187]. A 26-week aerobic exercise intervention significantly decreased plasma Cer levels in individuals at a high risk for AD, restoring Cer homeostasis [173]. Acute exercise transiently increased the S1P levels in blood and skeletal muscle [185,198], an effect potentially mediated by increased S1P release from erythrocytes [186]. In AD patients and MCI patients, 16 weeks of moderate-to-vigorous exercise and 24 weeks of multicomponent exercise, respectively, reduced the TC and LDL levels while increasing the high-density lipoprotein (HDL) levels in the blood [16,199]. Comparable results were observed in an AD animal model: three-month or 16-week treadmill exercise regimens reversed the abnormal TC, LDL, and HDL levels in the skeletal muscles and serum of AD mice, significantly reduced cerebral Aβ_42_ deposition, and improved cognitive function [189,190].

A single bout of prolonged vigorous exercise (75-km cycling competition) significantly increased the plasma LA levels in healthy adults [200]. Treadmill training for 6–7 weeks markedly elevated the LA concentrations in the plantar muscles and liver in Sprague–Dawley rats [201]. In contrast, a 24-week combined endurance and resistance training intervention significantly reduced the plasma LA levels in young sedentary adults [192]. Numerous studies have demonstrated that exercise reduces tissue TG levels. A meta-analysis indicated that various exercise modalities effectively decrease the hepatic TG content in patients with nonalcoholic fatty liver disease. Long-term aerobic exercise results in more pronounced TG lowering, whereas short-term or resistance training may have a limited effect [202]. Notably, a 16-week moderate-to-vigorous aerobic intervention failed to significantly reduce the plasma TG levels in AD patients [16]. However, a 24-week multicomponent intervention consisting of aerobic training, resistance exercises, and cognitive stimulation significantly decreased the plasma TG levels in older adults with MCI, concurrently reducing oxidative damage due to the systemic oxidative stress while improving physical fitness and cognitive function [199]. With respect to SM metabolism, 4 weeks of high-intensity training significantly decreased the plasma SM levels in healthy individuals [188], and acute exhaustive treadmill exercise reduced the SM content in the skeletal muscles of healthy Wistar rats [187]. However, neither acute exercise nor a 6-week treadmill training regimen significantly altered the total cardiac SM levels in healthy mice [184].

#### Potential mechanisms by which exercise influences AD through modulation of lipid homeostasis

Exercise can ameliorate Aβ pathology through regulation of lipid homeostasis via multiple pathways. Studies have demonstrated that a 4-week treadmill exercise regimen may upregulate ABCA1 expression, increase APOE lipidation efficiency, and promote Aβ degradation via NEP (neprilysin) and IDE [203]. A 12-week treadmill exercise intervention reduced Aβ deposition in the hippocampus of APP/PS1 mice by modulating the ADAM10 and BACE1 levels and decreasing cholesterol-mediated lipid raft formation [204]. Further research revealed that 12 weeks of aerobic exercise activated the AKT/SEC24D pathway, promoting the nuclear translocation of SREBP2, thereby promoting cholesterol homeostasis and ultimately suppressing the amyloidogenic cleavage of APP, leading to reduced Aβ generation [205]. A 5-month treadmill exercise protocol decreased the plasma TG levels in APP/PS1 mice, improved peripheral lipid metabolism in AD mice, and reduced hippocampal Aβ deposition [15]. As mentioned earlier, excessive Cer levels promote β-cleavage of APP and stabilize BACE1, thereby accelerating Aβ biosynthesis or facilitating Aβ deposition via exosome-mediated pathways [68]. Regular exercise inhibits Cer transport across the BBB, mitigates the Cer-induced interference with cerebral insulin signaling, prevents proinflammatory responses triggered by elevated Cer levels, and consequently reduces Aβ production, ultimately improving cognitive function [206]. Notably, exercise intensity can also modulate Aβ pathology through lipid pathways. Compared with low-intensity exercise, moderate-intensity exercise can more effectively ameliorate Aβ pathology through improvement of lipid metabolism and reduction of blood lipid levels [15].

Furthermore, exercise can modulate inflammation and oxidative stress through lipid regulation. A 6-week moderate-to-high-intensity treadmill exercise regimen reduced serum and skeletal muscle inflammation in male C57BL/6 J mice fed with a high-PA diet. This effect was mediated by activation of the SESN2/Nrf2 pathway, leading to decreased levels of proinflammatory cytokines such as TNF-α, IL-1β, and IL-6 in skeletal muscles [207]. An 18-week treadmill exercise intervention increased hepatic PC levels in a mouse model of liver tumor, thereby reducing the PA load and exerting anti-inflammatory effects [179]. Intriguingly, the exercise-induced myokine irisin has been demonstrated to effectively counteract the PA-induced metabolic disorders, lipotoxicity, and inflammation in multiple tissues, including the liver and skeletal muscle [208,209]. Exercise-induced increases in ARA levels ameliorate AD by increasing the levels of anti-inflammatory metabolites [210]. A 20-week long-term vigorous exercise regimen increased the TG levels in the gastrocnemius and soleus muscles of Sprague–Dawley rats. The elevated TG levels reduced ceramide accumulation, prevented skeletal muscle inflammation, and improved insulin sensitivity. The underlying mechanism may involve exercise-enhanced expression of SREBP1 (sterol regulatory element-binding protein 1) in skeletal muscle, thereby influencing the activity of TG synthesis pathways [174,211]. A 24-week multicomponent exercise intervention (including aerobic training, resistance training, and cognitive stimulation) significantly reduced the plasma TG levels in older adults with MCI, concurrently attenuating systemic oxidative stress and ultimately improving physical fitness and cognitive function [199].

In addition, the relationship between exercise and lipids is not one-directional. Improvements in individual lipid homeostasis can also enhance exercise capacity, thereby establishing a beneficial positive feedback loop that ultimately further mitigates AD-related pathological changes. On the one hand, exercise intervention enhances the beneficial effects of DHA. DHA promotes neurite outgrowth and synaptic membrane expansion by activating syntaxin-3 (STX-3) [212]. In Sprague–Dawley rats, 12 days of voluntary wheel running amplified the dietary DHA-induced upregulation of hippocampal STX-3, thereby facilitating synaptic growth, with a positive correlation between the levels of STX-3 and the amount of exercise. Moreover, the same exercise regimen amplified the increases of hippocampal NR2B and growth-associated protein (GAP-43) expression, enhancing synaptic plasticity and improving cognitive function. Notably, the amount of exercise was also positively correlated with hippocampal GAP-43 levels [213]. On the other hand, DHA supplementation attenuated tau phosphorylation at Ser422 and Ser396 by suppressing JNK activity in the brains of mice with traumatic brain injury, ultimately improving learning ability, memory deficits, and exercise function [117]. Combined aerobic exercise and DHA supplementation was also found to prevent gray matter volume loss in the frontal, parietal, and cingulate cortices of MCI patients, thereby mitigating AD-related brain atrophy [214]. Similarly, PL and exercise may exhibit bidirectional interactions. On the one hand, exercise may increase PL levels, in turn reducing oxidative stress, inhibiting ferroptosis, and activating the MAPK (mitogen-activated protein kinase) pathway, ultimately ameliorating AD pathology [215]. On the other hand, dietary PL supplementation improved swimming performance and increased the reaction speed of AD model zebrafish, leading to increases of swimming distance, swimming speed, and turning angles [215,216].

**Fig. 2:**
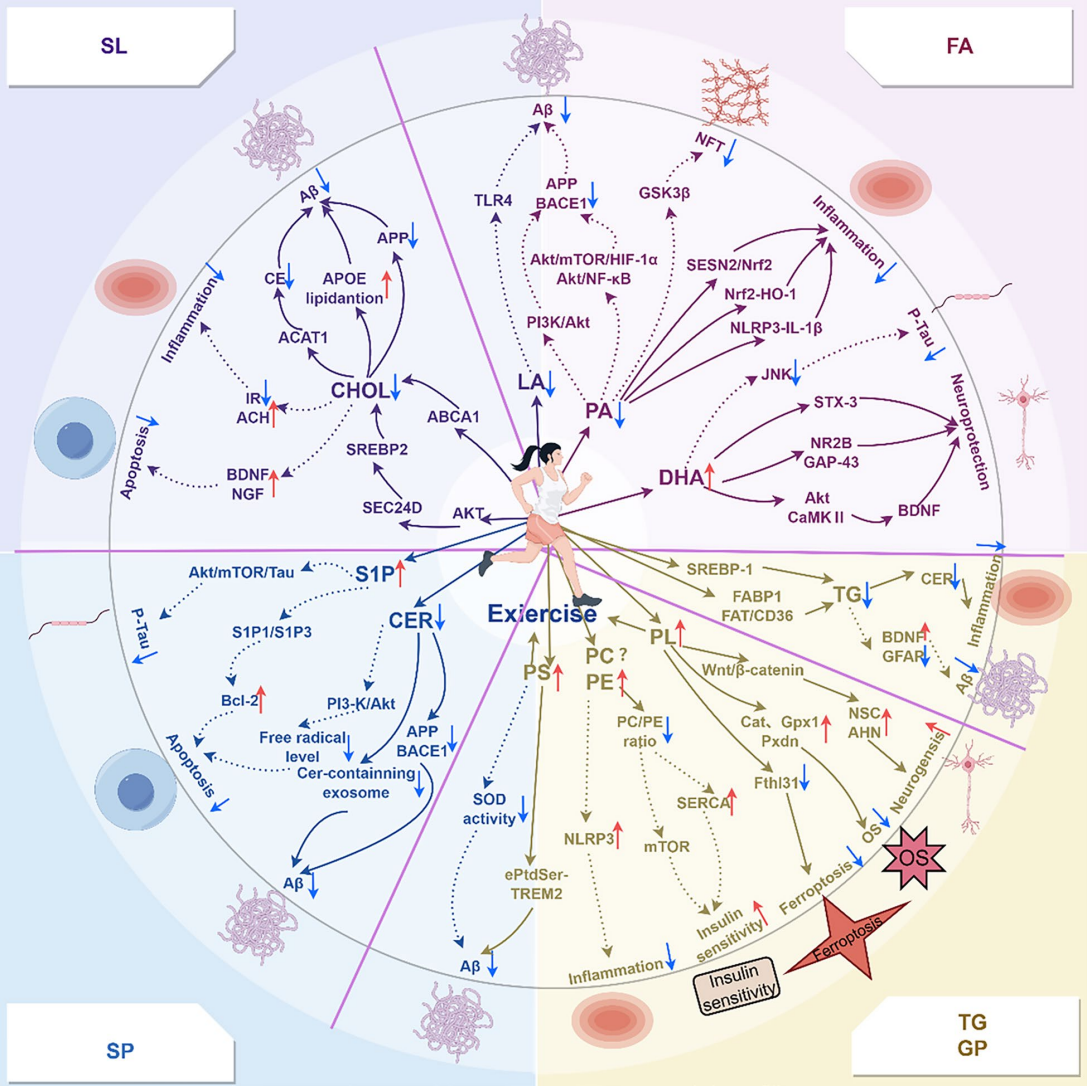
Molecular mechanisms by which exercise modulates the levels of multiple lipids and thereby affects AD. Solid lines in the figure indicate relationships that are supported by direct evidence, whereas dashed lines represent those inferred indirectly. Note: FA: fatty acid; LA: linoleic acid; TLR4: toll-like receptor 4; Aβ: amyloid-β; PA: palmitic acid; APP: amyloid precursor protein; BACE1: beta-site amyloid precursor protein cleaving enzyme 1; GSK3β:kinases GSK3β; NFT: neurofibrillary tangle; DHA: docosahexaenoic acid; JNK: c-Jun N-terminal kinase; STX-3: syntaxin-3; GAP-43: growth-associated protein; PI3K: phosphoinositide-3-kinase; CaMKII: Ca^2+^/calmodulin-dependent protein kinase II; BDNF: brain-derived neurotrophic factor; SREBP-1: element-binding protein-1; FABP1: hepatic fatty acid-binding protein 1; FAT/CD36: hepatic fatty acid translocase /CD36 proteins; TG: triglyceride; CER: ceramide; GFAP: glial fibrillary acidic protein; PL: plasmalogen; NSC: neural stem cell; AHN: adult hippocampal neurogenesis; Gpx1: glutathione peroxidase 1; OS: oxidative stress; PE: phosphatidylethanolamine; PC: phosphatidylcholine; SERCA: sarcoplasmic reticulum calcium ATPase; NLRP3:NOD-, LRR-and pyrin domain-containing protein 3; PS: phosphatidylserine; SOD: super oxide dismutase; S1P:sphingosine-1-phosphate; Bcl-2: anti-apoptotic-2; SEC24D: SEC24 homolog D, COPII coat complex component; ABCA1: ATP-binding cassette subfamily A member 1; CHOL: cholesterol; NGF: nerve growth factor; IR: insulin resistance; ACH: acetylcholine; CE: cholesteryl ester; ACAT1: Acyl-CoA: cholesterol Acyltransferase. Red arrows: level rises; Blue arrows: level decline; “?”: exists controversy

In summary, exercise modulates lipid homeostasis and exerts beneficial effects on AD through diverse biological pathways (Fig. 2). However, direct evidence linking exercise-induced lipid changes to AD improvement remains limited. Future studies should clarify whether specific lipid molecules directly mediate the exercise-induced amelioration of AD or merely represent epiphenomena. For instance, studies can use gene knockdown techniques to silence the genes encoding lipid metabolism-related enzymes in AD mouse models before assessing the effects of exercise in Aβ pathology. Subsequent intracerebroventricular replenishment of target lipids could be performed to determine whether the cognitive benefits can be reproduced independently of exercise.

## Lipids may act as mediators of the peripheral-central crosstalk between exercise and AD

**Fig. 3:**
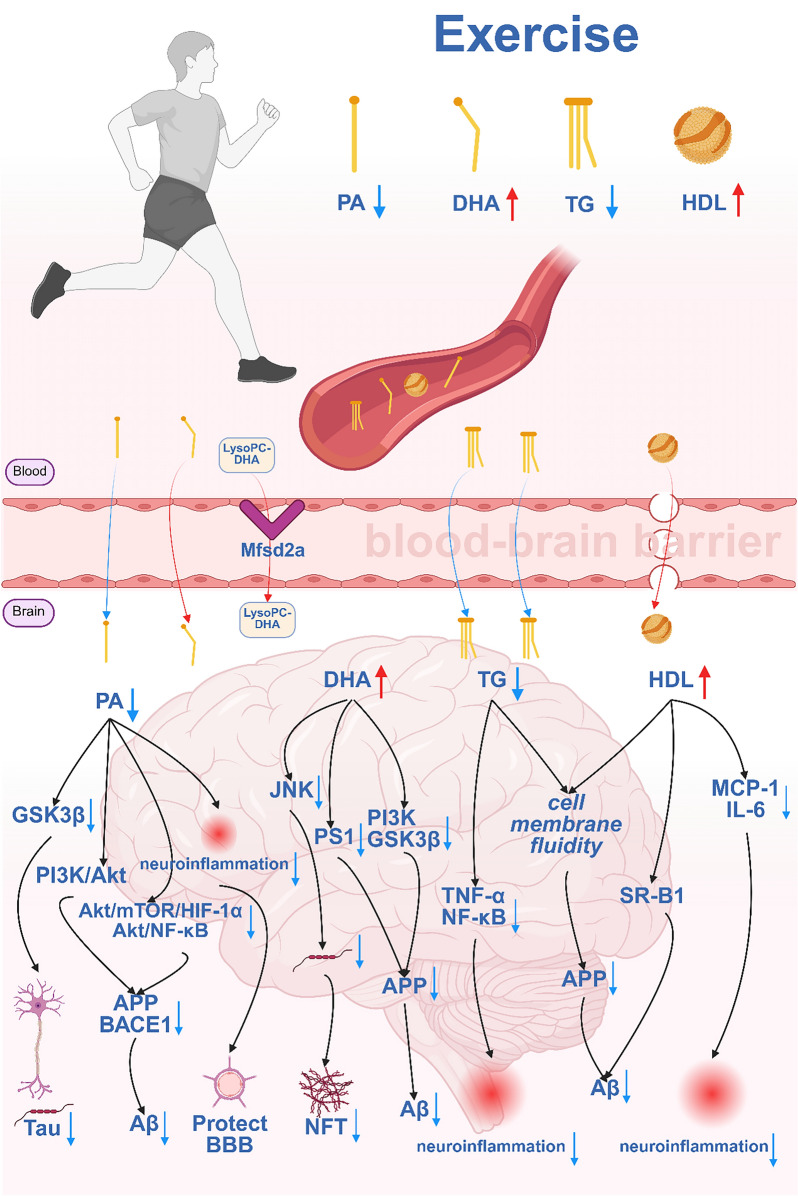
Mechanistic diagram of lipid-mediated peripheral-central crosstalk between exercise and AD. Note:Aβ, amyloid-β; Akt: protein kinase B; APP: amyloid precursor protein; BACE1: beta-site APP cleaving enzyme 1; BBB: blood–brain barrier; DHA: docosahexaenoic acid; GSK3β: glycogen synthase kinase 3 beta; HDL: high-density lipoprotein; HIF-1α: hypoxia-inducible factor 1 alpha; IL-6: interleukin-6; JNK: c-Jun N-terminal kinase; LysoPC-DHA: lysophosphatidylcholine-DHA; MCP-1: monocyte chemoattractant protein-1; Mfsd2a: major facilitator superfamily domain-containing 2a; NFT: neurofibrillary tangle; NF-κB: nuclear factor kappa-light-chain-enhancer of activated B cells; PA: palmitic acid; PI3K: phosphoinositide 3-kinase; PS1: presenilin 1; SR-B1: scavenger receptor class B type 1; TG: triglyceride; TNF-α: tumor necrosis factor-α

There is a strong association between exercise and AD, and epidemiological evidence shows that regular exercise significantly reduces the AD risk [226]. However, most of the studies on exercise and AD have focused more strongly on peripheral alterations. Due to the BBB and the inaccessibility of human brain tissue, the specific mechanisms underlying the central effects of exercise in AD patients are still unclear [13]. With recent advances in imaging and other technologies, some studies have found that exercise increases hippocampal volume [227]; however, these findings mainly reflect structural changes at the tissue level, and the specific underlying molecular mechanisms remain unclear. In this context, exploring potential factors that mediate   the peripheral–central link between exercise and AD may help explain how the peripheral effects of exercise influence the central nervous system. Recent studies have shown that some lipids can enter the brain through the BBB [228]. Isotopic tracer studies showed that lipids such as PA, DHA, OA, TG, and HDL can traverse the BBB via passive diffusion or transcytosis [229–233]. Notably, exercise regulates peripheral lipid metabolism [234]. Based on these findings, here we propose that lipids may act as mediators of peripheral–central crosstalk between exercise and AD. Specifically, we propose that exercise modulates peripheral lipid profiles, thereby influencing the central lipid homeostasis and ultimately conferring beneficial effects on AD-related pathology in the brain. Specifically, exercise reduces peripheral PA levels, inhibits the passive diffusion of peripheral PA into the brain, and thereby decreases cerebral PA levels. This decrease in cerebral PA levels subsequently decreases tau phosphorylation in neurons by suppressing the expression of GSK3β [103] (Fig. 3). Additionally, this decrease in cerebral PA levels downregulates the expression of APP and BACE1 through inhibiting the GPR40-mediated PI3K/Akt signaling pathway or suppressing the activation of Akt/mTOR/HIF-1α and Akt/NF-κB signaling, thereby reducing Aβ production [69]. Notably, elevated PA levels can induce inflammation and disrupt the BBB [235], whereas reducing PA levels suppresses inflammatory factors and protects BBB integrity [236]. Exercise increases peripheral DHA levels, facilitating the entry of DHA and LysoPC-DHA (lysophosphatidylcholine-bound DHA) into the brain via passive diffusion and Mfsd2a (major facilitator superfamily domain-containing protein 2A)-mediated active transport across the BBB, respectively [237,238]. The elevated cerebral DHA levels further inhibit tau phosphorylation at Ser422 and Ser396 through suppression of JNK activity [117]. Moreover, DHA can restrict APP secretase processing—ultimately decreasing Aβ production—by lowering presenilin-1 level and suppressing the PI3K and GSK3α pathways [79,80,239]. Exercise decreases peripheral TG levels, inhibiting the entry of TGs into the brain via passive diffusion or other potential pathways. This attenuates neuroinflammation by suppressing the expression of proinflammatory factors such as TNF-α and NF-κB [118]. Additionally, the reduction in TG levels may decrease Aβ production by modulating membrane fluidity and restricting the proteolytic processing of APP [240]. Exercise elevates peripheral HDL levels, enabling HDL to cross the BBB via receptor-mediated transcytosis. In the brain, HDL binds to apolipoprotein A-I (APOA-I) and promotes cholesterol efflux through ABCA1 or influences membrane fluidity, ultimately reducing Aβ production by limiting the proteolytic processing of APP [241,242]. Furthermore, cerebral APOA-I/HDL complexes may bind to Aβ and facilitate its clearance via scavenger receptors (e.g., SR-B1), thereby lowering Aβ levels in the brain [243]. Exercise may also mitigate neuroinflammation by reducing the production of proinflammatory chemokines/cytokines, such as MCP-1 (monocyte chemoattractant protein-1) and IL-6 [244]. The establishment of the “exercise–peripheral lipid–BBB–CNS lipid” axis helps clarify how exercise builds a molecular bridge between the periphery and the central nervous systems through lipids that can cross the BBB.

## Exercise, *APOE* and AD

The *APOE* ε4 allele is the strongest genetic risk factor for AD [245]. Interestingly, it may also influence the relationship between exercise and cognition. Longitudinal studies have found that physical activity reduces the risk of cognitive decline and hippocampal atrophy only in *APOE* ε4 carriers, while no significant associations were found in noncarriers [246,247]. However, due to the limitations of the studies, a causal relationship could not be established. A further RCT in AD patients revealed that aerobic training improved the cognitive performance of *APOE* ε4 carriers compared with the carriers without aerobic training, whereas there was no change in the *APOE* ε4 noncarriers [248]. Similarly, another RCT showed that the *APOE* ε4 carriers showed greater upregulation of BDNF following the intervention, suggesting that BDNF signaling may be one of the pathways mediating the cognitive improvement [249]. However, related studies have reported inconsistent results. For example, physical activity reduced the risk of AD in the *APOE* ε4 noncarriers but was not associated with a significant reduction in the *APOE* ε4 carriers in a longitudinal study with a mean follow-up of 5.4 years [250]. An RCT in younger adults reported improvement of executive function after aerobic training solely in noncarriers [251]. In another 24-week RCT, the *APOE* ε4 noncarriers but not the *APOE* ε4 carriers had significantly higher Alzheimer’s Disease Assessment Scale-Cognitive subscale (ADAS-Cog) scores after the intervention [252]. Most scholars have attributed the above discrepancies to the differences in the age and the number of participants, or the inconsistency in clinical diagnosis, a rationale that has some validity but is too broad in scope. Exploring how the age, the diagnostic criteria, or the number of participants may specifically affect the above disparities may be a direction for future research. In future studies, the scientific rigor and comparability of research could be increased by application of refined population stratification, standardized intervention protocols, and unified outcome measures. Additionally, conducting multicenter, large-scale prospective RCTs and integrating multiomics data would facilitate the systematic biological delineation of exercise-induced response patterns among individuals with different *APOE* ε4 genotypes. Furthermore, regardless of the effect of the *APOE* ε4 allele on the relationship between exercise and AD, the specific biological mechanisms through which exercise affects AD are similar. Exercise has been shown to ameliorate *APOE* ε4-associated neuropathology, such as inhibiting Aβ deposition, upregulating neurotrophic factors (e.g., BDNF), reducing neuroinflammation, and regulating glucose metabolism [253]. Further investigations should focus on mechanisms underlying the differential molecular responses (e.g., the responses of BDNF, Aβ, tau, and inflammatory markers) between *APOE* ε4 carriers and noncarriers following exercise interventions.

## Conclusions and future perspectives

In summary, abnormal levels of multiple lipids are involved in the development of AD. Numerous experimental studies have shown lipid dysregulation in the peripheral tissues and brains of AD patients and AD model animals. Lipid dysregulation is involved in the pathological process of AD through promotion of Aβ production, increase of tau hyperphosphorylation, and exacerbation of neuroinflammation and oxidative stress. As a nonpharmacological intervention, regular exercise can modulate lipid homeostasis in the periphery and brain, ultimately contributing to the amelioration of AD pathology by reducing the Aβ burden, attenuating neuroinflammation, enhancing neuronal activity, and inhibiting apoptosis.

This review highlights three major advantages of exercise intervention in regulating lipid homeostasis in AD. First, lipid homeostasis constitutes a vast and intricate process involving numerous lipid species with heterogeneous distributions throughout the body. These lipids have diverse biological effects, and their interconversion is dynamic and complex. As a holistic therapy, exercise has multitarget effects. It can comprehensively regulate lipid homeostasis and exert specific regulatory effects on different lipids, and its overall regulatory effect is more compatible with the complex characteristics of lipid homeostasis. Second, accumulating research indicates that the dysregulation of lipid homeostasis often occurs initially in the early stages of AD. Therefore, early lipid-centered therapeutic strategies may be effective in mitigating the progression of AD and help extend the therapeutic window. Moreover, individuals in the early stages of AD generally retain sufficient functional capacity to participate in structured exercise programs. Therefore, regular exercise is recognized as one of the optimal early-stage interventions for AD. Finally, while current lipid-targeting pharmacological therapies—such as statins and LXR agonists—have shown some promise in modulating lipid metabolism and alleviating AD-related pathology, they are often associated with significant and unavoidable side effects. In contrast, exercise interventions demonstrate a favorable safety profile and are cost-effective, and they may also help counteract the adverse effects of pharmacotherapies. In addition, we propose a hypothesis that lipids are potential factors mediating the peripheral–central crosstalk between exercise and AD. We also explore the effects of different *APOE* genotypes on the association between exercise and AD.

However, several limitations exist in research on the exercise-mediated regulation of lipid homeostasis in AD. First, although exercise plays a pivotal role in the systemic regulation of lipid homeostasis, current mechanistic studies exploring how exercise influences AD through specific lipid species remain relatively scarce. In particular, among lipid species, TGs have been shown to be closely associated with AD, but the mechanism by which exercise affects AD via TGs is unknown. This lack of knowledge about TGs hinders further in-depth investigations, such as those related to the development of lipid-specific drug targets. Elucidating the targeted mechanisms of individual lipid species would contribute to refining the comprehensive regulatory framework of lipid homeostasis. Additionally, while exercise effectively delays AD progression by modulating lipid homeostasis during early disease stages, its therapeutic efficacy may be limited once neurodegenerative changes have advanced beyond a certain threshold. In such cases, exercise may neither fully alleviate symptoms nor reverse disease progression, and a patient’s physical condition might preclude high-intensity vigorous exercise. Therefore, exercise should not only be considered a disease-modifying intervention, but also as a preventive strategy.

Finally, studies are needed to further explore personalized exercise regimens for AD prevention and treatment, including optimal exercise modalities, duration, and intensity, as well as the combined therapeutic effects of exercise with other nonpharmacological interventions. Given the distinct properties of various classes of lipids—for instance, the preferential modulation of cholesterol by statins, the predominant influence of dietary factors on fatty acids, and the regulation of glycerophospholipids by exercise—an integrative framework combining multiple strategies (e.g., pharmacological, dietary, and exercise interventions) should be established for AD prevention and treatment. This approach may provide additional intervention methods and theoretical foundations for AD management.

## Data Availability

Not applicable.

## Abbreviations

ABCA1: ATP-Binding Cassette a1
ACH: Acetylcholine
AD: Alzheimer’s disease
ALA: α-Linolenic acid
ALT: Alanine aminotransferase
APP: Amyloid precursor protein
ARA: Arachidonic acid
Aβ: Amyloid-β
BACE1: Beta-site amyloid precursor protein cleaving enzyme 1
BBB: Blood–brain barrier
BDNF: Brain-derived neurotrophic factor
C1P: Ceramide-1-phosphate
CE: Cholesteryl esters
Cer: Ceramide
CNS: Central nervous system
CSF: Cerebrospinal fluid
DHA: Docosahexaenoic acid
GPR40: G protein-coupled receptor 40
GPs: Glycerophospholipids
HDL: High-density lipoprotein
LDL: Low-density lipoprotein
LXR: Liver X receptor
MCTs: Medium chain triglycerides
NFTs: Neurofibrillary tangles
OA: Oleic acid
PA: Palmitic acid
PC: Phosphatidylcholine
PE: Phosphatidylethanolamine
PL: Plasmalogens
PS: Phosphatidylserine
ROS: Reactive oxygen species
S1P: Sphingosine-1-phosphate
SM: Sphingomyelin
SPs: Sphingolipids
TG: Triglycerides
TNF-α: Tumor necrosis factor-α
